# Risk analysis of biological hazards in food industry in terms of Public Health

**DOI:** 10.1093/eurpub/ckac131.231

**Published:** 2022-10-25

**Authors:** A Vantarakis, M Dimitrakopoulou

**Affiliations:** Medical School, University of Patras, Patras, Greece

## Abstract

Numerous pathogens are transmitted via food and according to recent surveys, there are millions of incidents recorded in Europe in the last decade. More specific, foodborne pathogens such as bacteria, fungi, parasites, and viruses can be detected in different stages of production or distribution of a food product. Examples of predominant symptoms caused by these foodborne pathogens are nausea, vomiting, diarrhea, cramps, fever, headache, cough etc. Therefore, it is essential need to detect, manage, and prevent of foodborne pathogens “from farm to fork” regarding consumers health risks. Europe has established and suggested management systems in food industry that control hazards in food products. However, outbreak incidents by foodborne pathogens existing until now. By using PRISMA guideline, we searched for the most recent publications referring microbiological risk assessments from online databases Scopus, PubMed and Science Direct. From 505 articles initially captured, data was extracted from 84 studies regarding microbiological risk factors in terms of food quality and safety, that are evidenced in European studies. Moreover, information about country of origin, food type, production phase and technology used for detection of pathogens, are also presented. Our results indicated that quality systems should be further developed to control all possible routes of contamination in the supply chain. This work provided information to managers in food industry and scientists for further research regarding microbiological risk assessments. Implementation of effective risk management systems in food industry could contribute to identify and eliminate potential risks and thus, consumer’s health and food quality could be reassured. Therefore, our findings could provide managers in food industry either to build up more effective management systems or even help scientists to better understand ecology of pathogens regarding food matrix and environmental conditions.

**Key messages:**

• Risk analysis of biological hazards in food industry could prevent foodborne diseases.

• Risk analysis of foodborne pathogens is important for public health.

